# DOES SOCIAL CAPITAL ENHANCE POLITICAL PARTICIPATION? A MULTILEVEL ANALYSIS OF YOUNGER AND OLDER ADULTS IN EUROPE

**DOI:** 10.1093/geroni/igad104.1675

**Published:** 2023-12-21

**Authors:** Fredrica Nyqvist, Mikael Nygård, Rodrigo Serrat, Marina Näsman

**Affiliations:** Åbo Akademi University, Vaasa, Pohjanmaa, Finland; Åbo Akademi University, Vaasa, Pohjanmaa, Finland; University of Barcelona, Barcelona, Catalonia, Spain; Åbo Akademi University, Vaasa, Pohjanmaa, Finland

## Abstract

Political participation encompasses institutionalised activities such as attending meetings of a political organisation as well as non-institutionalised activities including contacting politicians, signing petitions or boycotting. Both forms of activities tend to be dependent upon birth cohort, political socialisation, and period effects like changes in the welfare state design. Furthermore, it is also connected with other domains of civic engagement as well as trust. Social capital theory focuses on social connections and roles fostering civic engagement, including political participation, however, the relationship between social capital and political participation might look different for different types of political activities. The aim of this study was, therefore, to test the importance of social capital for different types of political participation in younger and older adults by analysing cross-national data from the European Quality of Life Survey (EQLS) in 2016. Data was collected in 33 European countries and includes more than 36,000 individuals aged 18 and over. Multilevel regression analysis was used to explore individual-level and country-level social capital on different measures of institutionalised and non-institutionalised political participation in younger and older adults. The results showed that a third of the sample participated in non-institutionalised activities whereas 11 percent participated in institutionalised activities. Both forms of political participation were lower among older adults as compared to younger age groups. While social capital was identified as an important predictor of political participation, it was also shown that these associations were conditioned by larger cultural, political and welfare institutional contexts.

